# A Case Report of Right Heart Failure: An Uncommon Presentation: A Case Report of Right Heart Failure

**DOI:** 10.31661/gmj.v14i.3921

**Published:** 2025-10-12

**Authors:** Venus Shahabi Rabori, Oliver McConnell, Olivia Powell

**Affiliations:** ^1^ International Training Fellow of Cardiology, Royal Albert Edward Infirmary of Wigan, Wwl NHS Trust; ^2^ Consultant Cardiologist, Macclesfield District General Hospital, East Cheshire NHS Trust; ^3^ Cardiac Physiologist, Royal Albert Edward Infirmary of Wigan, Wwl NHS Trust NHS Trust

**Keywords:** Aortic Dissection, Stanford Type A Aortic Dissection, Right Heart Failure, Coronary Vessels, Myocardial Infarction (as differential diagnosis), Computed Tomography Angiography

## Abstract

**Background:**

Aortic dissection poses diagnostic challenges due to its varied symptoms. Prompt diagnosis and intervention are essential to reduce mortality and morbidity.

**Case Report:**

A 53-year-old woman presented with dyspnoea, palpitations, Epigastric and right upper quadrant pain, following recent chest tightness. She initially was diagnosed with a non-ST elevation myocardial infarction (NSTEMI) based on the electrocardiogram (ECG) and cardiac enzymes. The transthoracic echocardiogram (TTE) showed right heart impairment, mild aortic regurgitation, and significant tricuspid regurgitation. A coronary angiogram showed normal left coronary vessels but failed imaging of the right coronary artery which raised suspicion of aortic dissection due to an abnormal aortic root shape and a history of hypertension. Urgent CT aortography (CTA) confirmed acute Stanford type A aortic dissection with false lumen supplying the RCA ostia. Initially, conservative management was chosen due to right ventricular dysfunction. The patient then presenting with recurring symptoms 8 days later, after a multidisciplinary team meeting surgical intervention was decided. Comprising of full aortic root, aortic arch, and aortic valve replacement, plus tricuspid valve repair and annuloplasty. The patient was discharged post successful surgery.

**Conclusion:**

This case highlights the challenges of diagnosing and managing acute aortic syndromes, especially with atypical symptoms. Having a low threshold for cross sectional imaging techniques in such cases is likely to prompt accurate diagnosis and treatment in critical cases.

## Introduction

Aortic dissection (AOD) is a serious cardiovascular condition marked by a complicated pathological process where the layers of the aortic wall separate [1][2]. Although AOD is rare, it presents significant challenges due to its high mortality rate [3]. Symptoms can be varying, resembling myocardial ischemia, and may include sudden chest or back pain characterized by a tearing sensation along with examination findings indicative of aortic regurgitation and mediastinal widening on chest X-ray [4][5]. Numerous conditions are associated with increased stress on the aortic wall that eventually give rise to AOD. Hypertension emerges as the most significant modifiable risk factor, with 75-80% of cases having a history of arterial hypertension [6][7].

Patients may face various complications, including heart failure, cardiac tamponade, neurological issues, fainting, and other symptoms of vascular insufficiency and malperfusion syndromes. Dissections can also involve the coronary arteries, with the right coronary artery being the most affected. Triple rule out computed tomography (TRO-CT) serves as a highly sensitive diagnostic tool for aortic dissection, especially in individuals presenting with acute chest pain who are at low to moderate risk for acute coronary syndromes (ACS) or pulmonary embolism (PE) [4][5][6][7][8].

## Case Presentation

**Figure-1:**
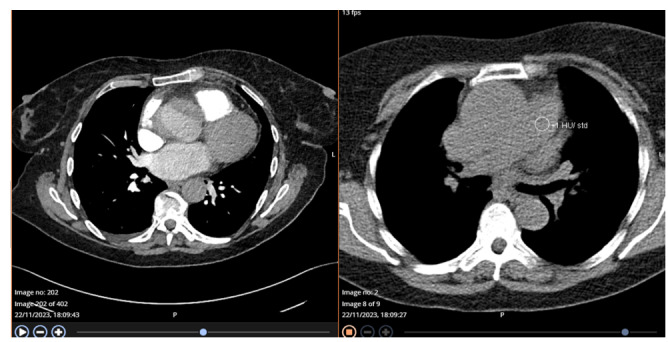
Figure 1. First CT Angiography to rule out pulmonary embolism (PE) showed dilated Ascending Aorta with a dissection flap that can be seen in the retrospective analysis and no evidence of PE.

**Figure-2:**
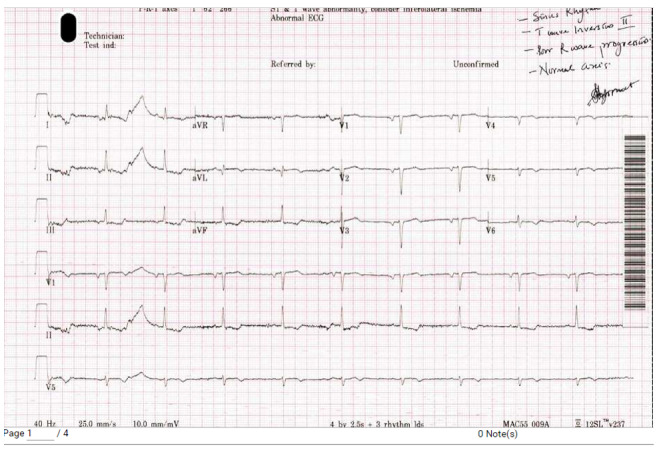
Figure 2. ECG on the first presentation showed ST depression in the inferolateral leads in addition to T wave inversion in precordial leads consistent with ischemia in the RCA territory.

**Figure-3:**
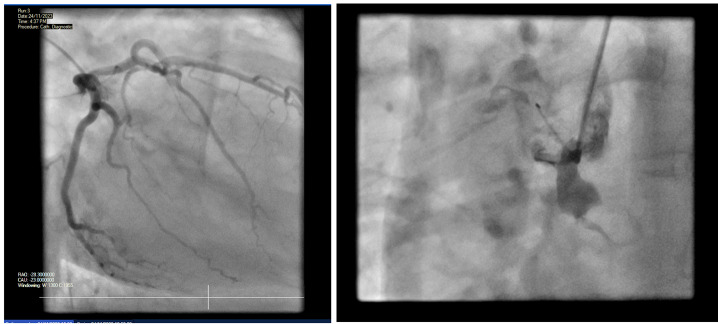
Figure 3. A: Coronary angiography on second day of first presentation showed normal/patent left main artery and diagonal branches. B: Coronary angiography on second day of first presentation showed abnormal shape of aortic root, unsuccessful RCA cannula

**Figure-4:**
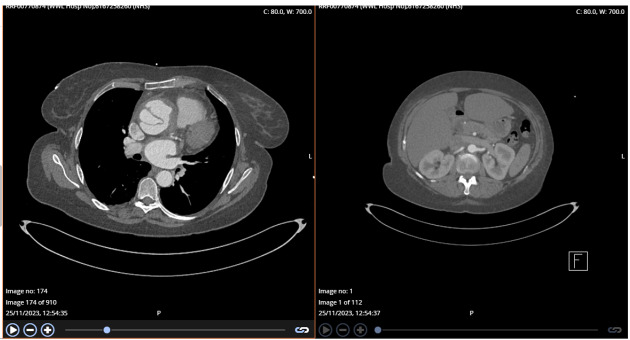
Figure 4. Abdominal and Aortic CT on second day of first presentation diagnosed Aortic dissection

**Figure-5:**
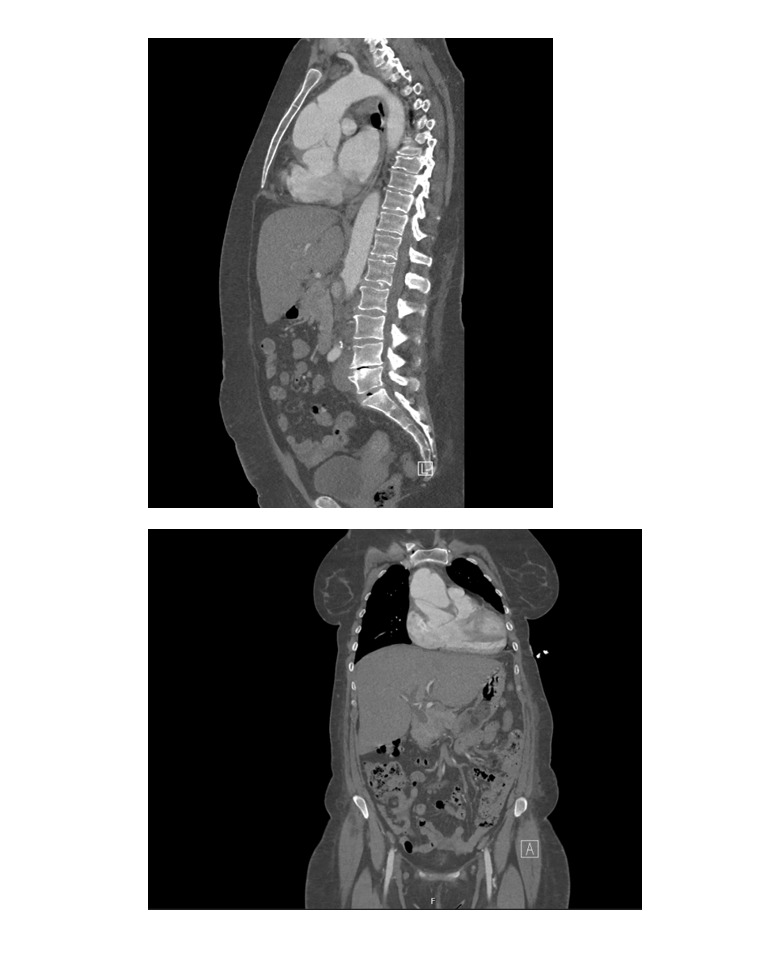
Figure 5. CTA whole Aorta at second presentation confirmed previously diagnosed aortic dissection

On November 22, 2023, a 53-year-old lady who had been treated with citalopram, propranolol, ramipril, and mirtazapine for more than five years for anxiety, depression, hypertension, and excessive smoking was admitted to the hospital. She was experiencing shortness of breath, abdominal pain, and palpitations. Physical examination revealed :BP=110/86 mmhg, PR=61, Sao2=99%, dyspnoea, a grade II murmur, and a tender epigastrium. Blood tests showed elevated CRP level at 214 mg/l, new-onset acute kidney injury ;evidenced by reduced kidney filtration rate and higher creatinine levels compared to results from two months prior; increased D-dimer at 1049ng/ml, and mildly elevated liver enzyme to 62U/L. Due to her shortness of breath and elevated D-dimer, the surgical team ordered a CT scan of the abdomen and pelvis, as well as a CT pulmonary angiogram.

The patient was treated with Tazocin and IV fluids and had a CT scan that showed dilation of the common bile duct (CBD), no visible stones, and fluid surrounding the gallbladder along with mild thickening (Figure-1-A). These findings were indicative of acute acalculous cholecystitis, along with unexpected cardiac issues, such as an ascending aortic aneurysm and cardiomegaly (Figure-1-B). Further evaluation shifted the focus to her cardiac conditions, as the CT results regarding the gallbladder could also be influenced by heart failure.

Elevated cardiac troponin levels, ST segment depression in leads I, II, III and T wave inversion in precordial leads raised concerns for ACS (Figure-2). Treatment commenced for NSTEMI. The TTE reported a hypertrophied left ventricle (LV) with normal systolic function >55%, a dilated left atrium (LA) and aorta, as well as a dilated right atrium (RA) and right ventricle (RV) with impaired systolic function. The patient underwent a coronary angiogram (Figure-3) revealing normal left coronary arteries however, it also indicated an abnormal aortic root and unsuccessful engagement of the RCA, raising concerns about a possible AOD.

A follow-up CTA performed the following day (Figure-4) confirmed a complex Stanford type A aortic dissection with concurrent aneurysmal dilatation, measuring 6.4 cm at its widest point and affecting both the ascending aorta and aortic root. Notably, the right coronary sinus and artery were supplied by the false lumen, which clarified why the initial attempt to engage the RCA during the angiogram was unsuccessful. The dissection flap ended just before the innominate artery, with all three major cranial vessels receiving blood from the true lumen. The rest of aorta appeared normal, with no signs of contrast extravasation, and only a small right pleural effusion was noted.

### Diagnosis and Management

Patient was diagnosed with type A AOD and was advised to undergo cardio-thoracic surgery. The case was discussed in several multidisciplinary meetings, where a careful risk-benefit analysis was conducted considering the timing of the initial presentation and the RV dysfunction. The patient was admitted to the intensive care unit for strict blood pressure management with labetalol up to SBP under110 mmhg and close monitoring. After ten days, she was discharged with a plan for outpatient follow-up, including a repeat TTE and CTA scheduled in four weeks.

However, eight days after discharge she returned to a local hospital with recurring symptoms of chest heaviness, shortness of breath, and palpitations.

An ECG was performed, revealing findings like those from her previous hospitalization. This admission resulted in a repeat CTA of the entire aorta (Figure-5), which showed that the ascending aortic dissecting aneurysm remained unchanged, measuring 6.4 cm at its largest dimension. The TTE showed normal LV size with borderline low normal function 50-54%, a hypokinetic septum, a dilated aortic root and ascending aorta with a dissecting flap, mildly dilated RV with impaired function, moderate tricuspid regurgitation, and mild aortic regurgitation.

The patient was admitted to the local hospital’s intensive care unit. for monitoring, and later transferred to a critical care unit at a tertiary centre hospital before undergoing surgery. A tertiary centre was chosen because they have the capability to provide RV support with ECMO if necessary .

Emergency type A dissection repair was performed five days after second presentation, including mechanical aortic valve replacement, root replacement, ascending aorta replacement, hemiarch replacement, and coronary artery bypass grafting to the right coronary artery.

Concurrent tricuspid valve repair was undertaken due to severe tricuspid regurgitation. The patient faced complications like respiratory failure, pulmonary oedema, pneumonia, delirium, and fluid overload. Management strategies included respiratory support, antibiotic therapy, and addressing electrolyte imbalances.

After spending over three weeks in the critical care unit, the patient finally moved to a post-operative ward. A follow-up CTA imaging the day before discharge revealed a satisfactory condition of the aortic root, ascending aorta, adjacent arch, and tricuspid valve.

## Discussion

The case highlights diagnostic challenges of AOD, particularly its potential to mimic acute MI [1][5][9]. Also, AOD may infrequently lead to MI, which may raise mortality rates in this population [9]. A heightened level of clinical suspicion for AOD is crucial to avoid the administration of inappropriate medical treatments [5]. In this instance, the patient's vague symptoms, with history of anxiety/depression and hypertension, led to missed diagnosis and unnecessary coronary angiography during the initial two days of hospitalization which could resulted in serious consequences.

Management required a multidisciplinary approach involving cardiology, cardiothoracic surgery, and critical care. Despite initially adopting a conservative strategy, surgical intervention was conducted, addressing acute dissection and cardiac pathology. The patient's right ventricular function impairment and ventilation issues complicated perioperative management [10].

Open surgical repair is the standard treatment for proximal aortic dissections, while endovascular interventions are recommended for distal or type B dissections, with a 10-year survival rate of 30-60%. [3][11][12]

This patient experienced post-operative complications after a successful surgery, requiring a tailored approach to effectively manage these risks. Follow-up recommendations emphasise a structured approach with regular assessments and potential complication surveillance.

## Conclusion

In conclusion, this case study underscores the importance of maintaining a high index of suspicion, utilizing advanced imaging techniques, and adopting a comprehensive approach in managing complex cardiovascular conditions.

## Conflict of Interest

None.
